# Preoperative radiotherapy for rectal cancer: a comparative study of quality control adherence at two cancer hospitals in Spain and Poland

**DOI:** 10.2478/raon-2014-0008

**Published:** 2014-04-25

**Authors:** Magdalena Fundowicz, Miguel Macia, Susanna Marin, Marta Bogusz-Czerniewicz, Ewelina Konstanty, Ignaci Modolel, Julian Malicki, Ferran Guedea

**Affiliations:** 1 Department of Radiotherapy, Greater Poland Cancer Centre, Poznan, Poland; 2 Department of Radiotherapy, Catalan Institute of Oncology, L’Hospitalet de Llobregat, Spain; 3 Department of Training, Scientific Cooperation and Quality Assurance, Greater Poland Cancer Centre, Poznan, Poland; 4 Department of Medical Physics, Greater Poland Cancer Centre, Poznan, Poland; 5 Department of Catalan Institute of Oncology, L’Hospitalet de Llobregat, Spain; 6 Department osf Electroradiology, University of Medical Sciences, Poznan, Poland

**Keywords:** clinical audit, quality control, preoperative radiotherapy

## Abstract

**Background:**

We performed a clinical audit of preoperative rectal cancer treatment at two European radiotherapy centres (Poland and Spain). The aim was to independently verify adherence to a selection of indicators of treatment quality and to identify any notable inter-institutional differences.

**Methods:**

A total of 162 patients, in Catalan Institute of Oncology (ICO) 68 and in Greater Poland Cancer Centre (GPCC) 94, diagnosed with locally advanced rectal cancer and treated with preoperative radiotherapy or radio-chemotherapy were included in retrospective study. A total of 7 quality control measures were evaluated: waiting time, multidisciplinary treatment approach, portal verification, *in vivo* dosimetry, informed consent, guidelines for diagnostics and therapy, and patient monitoring during treatment.

**Results:**

Several differences were observed. Waiting time from pathomorphological diagnosis to initial consultation was 31 (ICO) *vs.* 8 (GPCC) days. Waiting time from the first visit to the beginning of the treatment was twice as long at the ICO. At the ICO, 82% of patient experienced treatment interruptions. The protocol for portal verification was the same at both institutions. *In vivo* dosimetry is not used for this treatment localization at the ICO. The ICO utilizes locally-developed guidelines for diagnostics and therapy, while the GPCC is currently developing its own guidelines.

**Conclusions:**

An independent external clinical audit is an excellent approach to identifying and resolving deficiencies in quality control procedures. We identified several procedures amenable to improvement. Both institutions have since implemented changes to improve quality standards. We believe that all radiotherapy centres should perform a comprehensive clinical audit to identify and rectify deficiencies.

## Introduction

In recent years, interest in improving the quality and efficiency of cancer care delivery has become increasingly urgent as health care costs have surged along with increased demand from an aging population. In 1999, a report entitled “Ensuring Quality Cancer Care” published by the Institute of Medicine in the United States described numerous quality control issues in cancer care.^1^ A major recommendation of the report was the need to establish a system to measure and monitor quality of care through the use of core set of indicators. In Europe, the European Union published a directive requiring that the implementation of clinical audits to improve quality in radiation medicine.^2^,^3^ Quality assessment through a system of indicators is still a relatively recent practice in radiotherapy and there is limited published research in this area. However, two recent studies in Italy, one by Cionini *et al*. and another by *the Instituto Superiore di Sanità* have attempted to establish quality indicators for radiotherapy.^4^,^5^

Given the relative paucity of quality control studies in radiotherapy, we decided to carry out a clinical audit of preoperative rectal cancer treatment at our two institutions, the Greater Poland Cancer Centre (GPCC) in Poland and the Catalan Institute of Oncology (ICO) in Spain.

The aim of this study was to select a set of relevant quality control measures and then determine institutional adherence to these standards in order to improve quality at our own institutions.

## Methods

We elected to use colorectal cancer to perform this clinical audit for 3 reasons: 1) In Europe, colorectal cancer is among the most common cancers, accounting for 436,000 cases (13.6% of the total) 6; 2) Both institutions in this study treat large numbers of patients for this disease; 3) Given the high incidence and mortality rates for rectal cancer, any improvements in our quality control procedures would have a large positive impact on a large numbers of patients. All of these factors made rectal cancer treatment an ideal process for comparison.

### Selection of quality indicators and standards

The aim of any quality assessment is to provide feedback on meaningful and interpretable measures, including cost-effectiveness.^7^,^8^ Cionini *et al.* recently performed a review of the scientific literature, including guidelines and national regulations, to select quality indicators based on the scientific literature to assure the highest strength of evidence. For this reason, we selected from the quality control indicators described by Cionini, choosing those most relevant to colorectal cancer. The following indicators were selected: 1) waiting times; 2) multi-disciplinary treatment approach; 3) portal verification; 4) informed consent; 5) *in vivo* dosimetry; 6) diagnostic & therapeutic guidelines; and 7) monitoring & review of patient during treatment. Table 1 provides a description of these indicators.

### Clinical audit

The clinical audit was performed as a 5-step process. First, each institution selected a multidisciplinary quality evaluation team consisting of departmental staff with experience in quality assurance. The 4-person GPCC team was composed of 1 radiation oncologist, 2 medical physicists, and 1 quality manager. The 4-person ICO team included 2 radiation oncologists (one of whom was assigned the role of “quality manager”), 1 medical physicist, and 1 statistician.

We used the International Atomic Energy Association (IAEA) QUATRO questionnaire as a model for our own modified survey.^6^ A checklist was created to organize the audit program and to ensure coverage of all relevant topics. To ensure objectivity, the 2-day on-site clinical audits were performed exclusively by the visiting institution’s team, who collected all necessary data on current local practices. All clinical audits were performed in the year 2008 by a 2- or 3-person team consisting of one radiation oncologist (two in the case of the ICO) and a medical physicist. The working language of the audit was English.

The clinical audit was performed as follows: a) audit preparation (appointment of auditing team, review of the background information prepared by the institution to be audited, and preparation of the audit program); b) entrance briefing: to introduce the auditors to the various staff members and to discuss the methods, objectives and details of the audit; and c) assessment: on-site clinical audit.

During the audit, staff members were interviewed about work practices and approaches, the facilities were inspected and all procedures and relevant documentation (including treatment records of the rectal cancer patients included in the study) were reviewed. In addition, the auditors observed directly the practical implementation of working procedures during the 2-day audit, including as many aspects of the patient treatment process (initial patient examination, diagnosis, evaluation, staging, treatment planning and delivery, and follow up) as feasible. An exit briefing was performed to give the host institution preliminary feedback.

### Patients and treatments

Patient inclusion criteria were as follows: locally advanced cancer of the middle and lower rectum diagnosed and treated with preoperative radiotherapy or radiochemotherapy during the year 2008. A total of 162 patients were evaluated (ICO=68; GPCC=94). All patients at the ICO underwent preoperative radiochemotherapy, as did 9 of the 94 patients from the GPCC; the remaining 85 GPCC patients received preoperative radiotherapy alone. All patients at both institutions were clinical stage T3/4 N−/+.

Preoperative radiotherapy was delivered using a high-energy linear accelerator (18 or 20 MV) with multileaf collimators and 3D treatment planning. All patients were treated in prone position with full bladder. At the GPCC, most patients were treated with a belly board for small bowel displacement. Three field technique to 25 Gy (5 Gy per day) at the GPCC or 45 Gy in 25 fractions (1.8 Gy per day) followed by a boost of 5.4 Gy in 3 fractions at the ICO and GPCC (9 patients).

Surgery was performed 6–8 weeks after completion of combined radiochemotherapy (ICO) or during the first week after completion of preoperative radiotherapy (GPCC). The definitive surgical technique included low anterior resection, abdominalperineal resection (Miles technique), Hartmann resection or tumour excision. Most patients at both institutions underwent surgery following completion of radiotherapy.

Portal imaging was performed on the first day of treatment at both institutions. Following protocol, in most cases (85 patients) treated at the Polish centre, only one portal check was necessary due to the short duration of radiotherapy (5 days). In patients who underwent combined radiochemotherapy, portal checks were performed every 10 days (and prior to the boost) at both institutions due to the longer treatment duration.

Clinical protocols at both institutions call for a deviation no greater than 5 mm. If deviations were considered excessive, corrections were made and the portal repeated. ICO performs either electronic or film portal depending on the accelerator, whereas only electronic portal imaging is available at the GPCC. The portal images are evaluated by a physician at the ICO while either a physicist or technician performs this function at the GPCC. Documentation from portal verification is available on the hospital network at the ICO or in the patient treatment chart (GPCC).

*In vivo* dosimetry was offered only at the GPCC, where it is performed in all cases before the first session, in the middle of the radiotherapy course, after treatment plan change, or on request of physician or physicist. Deviations between measured and expected dose were considered acceptable when they were less than 5% for open fields and 7% for wedged fields.

### Indicators

We evaluated seven different indicators (some with sub-indicators), described in Table 1.

### Statistical analysis

This was primarily a descriptive study. Quantitative data were analyzed using the Statistica PL 8.0 Statistical Software Package (StatSoft, Poland). Since numerical variables did not follow normal distribution, comparison between the two groups was performed using the Mann-Whitney U-test. The results of numerical data were expressed as median and range. Qualitative data were analyzed using Chi-square test of independence or, in cases in which zero observed frequencies occurred, the Fisher exact test. Statistical significance was established at *p*<0.05 in all the analyses.

## Results

### Indicator #1. Waiting times and compliance to treatment duration

#### Waiting time from pathomprphological diagnosis to initial consultation

The average length of waiting time for this indicator was 31 days at the ICO *vs*. 8 days for the GPCC, a significant difference (p<0.0001) Medians and range were 28.5 [87] and 5 [36] for ICO and GPCC, respectively.

#### Waiting time from the first visit to start the treatment

The average waiting time at the ICO is twice that (18 [53] *vs*. 8 [53]) of the GPCC, a significant inter-institutional difference (p<0.0001).

#### Existence of protocol for unplanned curative treatment interruptions

At the time of the audit, only the ICO had a protocol in place to compensate for unplanned interruptions of curative radiotherapy. The GPCC had no guidelines and compensation was *ad hoc* on a case by case basis.

#### Compliance to the prescribed overall treatment time: treatment interruptions

Most (85 of 94) GPCC patients underwent radiotherapy alone (without chemotherapy). As a result, the total radiotherapy treatment duration was 5 days (1 fraction/day), and no interruptions were recorded in these 85 patients. In contrast, treatment delivery time was considerably longer in the patients who underwent combined chemoradiotherapy (all 68 patients at the ICO and 9 patients at the GPCC). In these cases, radiotherapy consisted of 25 or 28 fractions delivered over a 33-day period. Of the 68 ICO patients who underwent combined chemoradiotherapy, 56 (82%) experienced treatment interruptions (mainly due to toxicity, machine malfunctions, holidays or unplanned quality control checks) versus 0 out of 9 cases (0%) at the GPCC. This large difference (82% *vs*. 0%) is notable but should not be considered significant given the small sample size (only 9 cases) at the GPCC.

### Indicator #2. Existence of a multidisciplinary treatment approach

At the time of study, the ICO had an established protocol for reviewing complex cases at a weekly interdisciplinary tumour board. No such protocol was in place at the GPCC. As per the ICO protocol, cases considered standard were not referred to the tumour board. Of the 68 patients at the ICO, 44 were referred to the interdisciplinary tumor board, while 24 (35%) were not.

### Indicator #3. Portal verification

#### Existence of protocol/recommendations for periodic verification of treatment fields

Both institutions had a protocol in place at the time of the study. Portal imaging is performed at both institutions on the first day of treatment. In the 85 patients who underwent radiotherapy alone at the GPCC, only one portal check was performed due to the short treatment time (1 fraction/day for 5 days). Portal checks for patients undergoing chemoradiotherapy were performed every 10 days at both institutions due to the longer treatment duration (25 fractions over 33 days). Portal checks were also performed before the boost.

#### Number of portal verifications per preoperative course of radiotherapy

Table 2 shows the number of patients for whom portal images were considered acceptable, unacceptable, or not performed. If deemed unacceptable due to deviations greater than those set by the protocol, the image was corrected and portal verification was repeated.

### Indicator #4. Informed consent & additional explanatory material

#### Informed consent

After verifying patient records, we found that the informed consent form was available for all GPCC patients whereas at the ICO, the signed forms were missing in 7 cases, a significant inter-institutional difference (p=0.0019).

#### Existence of a form, booklets, films and other supporting materials

Both institutions provided additional, detailed information. In the case of the ICO, detailed, specific information about the treatment was included in the informed consent form. At the GPCC, the patients were given a brochure with general information and a video with more detailed information.

### Indicator #5. In vivo dosimetry

#### Existence of a protocol and its content/recommendations for checking the entrance dose

At the time of study, only the GPCC had a protocol in place (as required by Polish law). *In vivo* dosimetry is not used for rectal cancer at the ICO.

#### Number of verifications (in vivo dosimetry) per pre-operative course of radiotherapy

*In vivo* dosimetry was performed on the 2^nd^ or 3^rd^ day of the treatment at the GPCC. Of the 94 patients at the GPCC, *in vivo* dosimetry was considered acceptable in 80 cases and unacceptable in 14. In these 14 cases, the dosimetry was recalculated to reach acceptable levels.

### Indicator #6. Guidelines for diagnostics and therapy

The GPCC follows the National Comprehensive Cancer Network (NCCN) guidelines for diagnosis and treatment of colorectal cancer.^9^ In contrast, the ICO uses locally-developed guidelines based largely on international guidelines (including the NCCN), but with some modifications as established by the hospital tumor board.

At both institutions, patients undergo digital rectal exam to determine eligibility for surgery. Subsequent assessment may include (depending on the institutional protocol) the following: colonoscopy with tumour biopsy, physical examination, chest and abdominalpelvic CT, chest X-ray, pelvic MRI, endoscopic ultrasound, and blood tests. All GPCC patients underwent x-ray examination versus 50% of patients at the ICO.

### Indicator #7. Review of rectal cancer patients during the treatment

At both institutions, patients receiving radiotherapy are reviewed once a week by a radiation oncologist. If problems are found, additional examinations/reviews are possible. This review process is governed by the protocols in place at both institutions.

## Discussion

The results of this study reveal that both institutions had some deficiencies in adherence to the quality indicators chosen for preoperative radiotherapy for rectal cancer. These findings confirm the need for independent audits in radiotherapy to identify deviations from good practice and to harmonize and determine what good practice is. In the paragraphs that follow, we contextualize our findings for each of the 7 indicators evaluated.

### Waiting times

Waiting times at the ICO were significantly longer for both variables (time from pathological diagnosis to first visit, and time from first visit to treatment). The wait from the first visit with the radiation oncologist to treatment at the ICO was double that of the GPCC. The reasons for these variations are many, but we suspect that main difference is that the sources of patient referrals to the ICO are much more heterogeneous than at the GPCC. In Spain, staging is performed by the radiation oncologist so that if any additional tests need to be requested, the start of treatment will be delayed. In many countries (including Poland), staging is performed before the patient is referred to the radiation oncologist; as a result, treatment can begin sooner.

Waiting times are perhaps among the most important quality variables and it is well-known that excessive waits can impact the results of treatment.^10^–^12^ Clearly, the main risk of increased waiting times is the possibility of tumour growth and metastasis.

Although no standard waiting times have yet been established, the National Health Service of the United Kingdom published a cancer plan that called for waits of no more than 1 month between referral by the general practitioner to the start of treatment.^13^ We believe, based on our experience and a literature review, that an optimal maximum waiting time should not exceed 21 days from first visit to start of treatment. Using these guidelines, both the ICO and the GPCC treated the patients in a timely manner, despite the large differences between the two institutions.

### Treatment interruptions

Treatment interruptions are another important indicator of quality that can have a marked effect on outcomes.^14^–^16^ The usual causes of interruptions include machine malfunction or maintenance, toxicity, holidays, or unplanned quality control checks. In such cases, it is essential to have a protocol in place to compensate for the interruption. At the time of our study, the ICO had a well-established comprehensive protocol to guide compensation for unplanned interruptions of curative radio-therapy. The GPCC had no established protocol at the time of the audit, although that has since been remedied.

We found important differences between the two hospitals in treatment interruptions, mainly because there were none at the GPCC. The GPCC patients received a short-course of radiotherapy (5 fractions in 5 days), while all 68 of the ICO patients (and only 9 GPCC patients) underwent an extended course of 25 fractions delivered in 33 days. For this reason, we can only reasonably compare results from the two groups who underwent a similar radiotherapy schedule. Unfortunately, the number of GPCC patients who received this schedule was too small (9 patients) for meaningful comparisons. Nevertheless, it is important to note that 82% of patients at the ICO experienced a treatment interruption. We know that treatment interruptions are a common occurrence in radiotherapy. This indicates an important quality control issue (poor record-keeping) that needs to be resolved.

In terms of this indicator, both institutions were found wanting: the GPCC for lack of a written protocol, and the ICO for failing to record the cause and responses to interruptions.

### Multidisciplinary approach

In recent years, more and more medical societies and institutions have come to accept the importance of using a multidisciplinary approach in cancer care to provide patients with optimal treatment for their specific characteristics.^17^–^20^ In this audit, we found that 35% of patients at the ICO were not presented to the board. However, this does not necessarily indicate a quality failure because the in-house protocol states that only unusual or complicated cases need to be brought to the board. Therefore, we must assume that those 35% of patients were considered standard cases (i.e., no unusual conditions). However, steps must be taken to assure, in the future, that this information is added to the patient records.

The GPCC was considered deficient in this category because no interdisciplinary treatment board was in place at the time of this audit. Fortunately, in this case the audit served its purpose, as the GPCC instituted a multidisciplinary approach in 2009.

### Portal verification

Portal Film or EPID (Electronic Portal Imaging Detection) is commonly used to evaluate the accuracy of the patient’s set up and of the field shape and geometry with respect to the treatment plan. Repeated verifications during treatments are aimed at controlling the stability and the reproducibility of treatment conditions. The relevance of this procedure varies for different treatments and consequently it is suggested to stratify the standard in relation to the treatment objective.

Our results showed that 3 patients at the ICO did not have a portal verification. The reason for this is not clear, but may be simply due to a failure to record the values in the patient records. However, this is a clear quality control failure that must be rectified. In contrast, all GPCC patients had the necessary verifications and adjustments when necessary.

### Informed consent

At the ICO, there were 7 cases in which we were unable to locate the informed consent form (versus no cases at the GPCC). While it is possible that these forms were never collected, we suspect that they were simply misplaced or lost.

In both countries the law requires that patients receive and sign an informed consent form prior to treatment. This is a basic patient protection method used in most countries.^21^ However, the fact that a patient signs the form does not necessarily mean he/she understands the treatment, as many patients will readily sign any document presented by the physician. One particularly shocking example of this was described by Byrne *et al*., who found that of 100 patients who underwent surgery, 27 did not know which organ had been treated.^22^ For this reason, we believe that patients should be given the information in a variety of formats, in addition to the legally required informed consent form. Aside from the informed consent form and a verbal explanation from the physician and/or nurse, we recommend that patients be given user-friendly brochures and videos that explain the treatment in an easy to understand way. We believe that our institutions should attempt to standardize the provision of information to patients, especially in providing written, treatment-specific information about the procedure and its expected outcomes and side-effects.

### *In vivo* dosimetry

*In vivo* dosimetry is a technique that uses semiconductors to ensure the calculated and measured doses are similar. It is used mainly for complex techniques and is useful for detecting rare cases of over- or underdosing.^23^,^24^ Although the routine use of *in vivo* dosimetry to prevent dosing errors may seem to be an obvious quality control measure, many centres do not use it. At present, *in vivo* dosimetry is not considered standard because there are doubts about its costs, time requirements, and clinical role, particularly in certain cancer localizations.^25^–^27^ For this reason, many centers prefer not to use it for routine procedures, especially because modern linear accelerators are believed to be more reliable and accurate than older ones. However, several authors continue to insist on the importance of *in vivo* dosimetry, notably Williams and McKenzie, who wrote an impassioned plea for its generalized use.^28^ However, we agree with the conclusions of a study by the Royal College of Radiologists in the UK, which stated that while *in vivo* dosimetry should be used at the beginning of treatment for most patients, each department should develop its own protocol.^29^ For the moment, the differences observed between protocols at our two institutions serve to illustrate the debate about the benefits of *in vivo* dosimetry for this localization and technique.

### Guidelines for diagnostics and therapy

Few would argue about the value of clinical guidelines provided that these have been prepared by expert groups and based on the best available evidence and practices. The benefit are many, as guidelines serve to standardize best practices, guide less-experienced physicians, and use evidence-based strategies.^30^,^31^ The ICO prefers to use locally-developed guidelines, which are based on and similar to international guidelines such as the NCCN. After performing this audit and seeing the merits of using guidelines tailored for a specific population and resources, staff at the GPCC began to develop their own local guidelines.

### Feedback to project partners

We performed this audit with a number of objectives in mind. The first and most important was to improve quality at our institutions. By identifying deficiencies in our processes, we hoped to eliminate these and so improve our results. We also wanted to contribute to the establishment of quality control indicators for radiotherapy and for a standard audit process.

Upon completion of the audit, both audit teams drafted a report of their findings. The results were discussed at a joint meeting, during which we discussed the deficiencies and agreed on joint standards based on the results of our clinical audit and a literature review.

To close the audit cycle, each institution began the process of implementing the newly-agreed standards. The intention is to perform a second audit in the future to verify the actual results of this process and to determine the effectiveness and usefulness of the new standards and improvements.

This was a retrospective study with a relatively small sample. Moreover, for both institutions, this was our first experience in performing a clinical audit for quality control.

## Conclusions

We believe that external audit programs such as ours can help to improve both patient safety and quality of care and this is why the IAEA has called for the development of comprehensive quality control programs for radiotherapy.^32^–^35^ However, there is still a glaring lack of experience in radiotherapy.

Performing a clinical audit is a time-consuming and labour-intensive process. However, despite the time and expense involved, the results have more than compensated the efforts. As a result of this study, both institutions have benefitted as we have identified numerous areas to target for improvement, which we hope will lead to better quality treatments and results. Moreover, the procedures developed here for rectal cancer can be adapted to improve treatment of other tumour localizations.

Our experience has also shown us that the road ahead will not be easy. Even in colorectal cancer, in which treatment is generally quite standardized, we still found a large gap between two similarly structured European hospitals. We believe that an independent external clinical audit is an excellent method of identifying and rectifying deficiencies in quality control procedures.

## Figures and Tables

**FIGURE 1.**
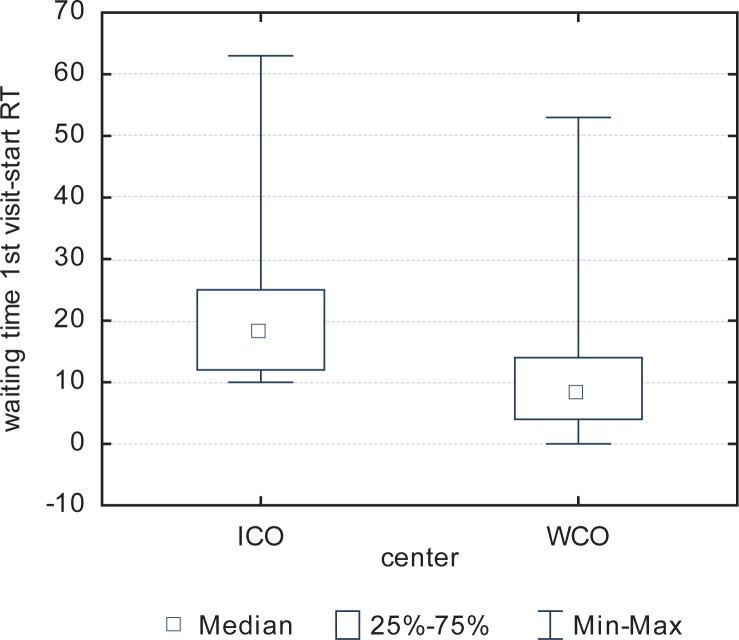
Waiting time from the first visit to start the treatment. RT = radiotherapy

**TABLE 1.** Quality control indicators evaluated

**#1: Waiting time and compliance**
1.1 Time from pathological diagnosis to initial consultation.
1.2 Time from the first visit to start the treatment
1.3. Existence of protocol for unplanned curative treatment interruptions
1.4 Compliance to the prescribed overall treatment time.

**#2: Existence of a multidisciplinary treatment approach**

**#3: Portal Verification**
3.1 Existence of protocol for periodic verification of treatment fields
3.2. Number of portal verifications per preoperative course of radiotherapy

**#4: Informed consent**
4.1 Existence of signed consent form in patient records
4.2 Availability of detailed information about treatment & side-effects

**#5. In vivo dosimetry**
5.1 Existence of protocol and recommendations for checking the entrance dose
5.2 Number of in vivo dosimetric verifications per preoperative course of radiotherapy.

**#6: Guidelines used for diagnostics and therapy.**

**#7. Monitoring & review of rectal cancer patients during the treatment period.**

**TABLE 2.** Portal imaging

	**Acceptable N (%)**	**Unacceptable N (%)**	**Not performed N (%)**
ICO	35 (51%)	31 (46%)	2 (3%)
GPCC	78 (83%)	15 (16%)	1 (1%)

N = number; ICO = Catalan Institute of Oncology; GPCC = Greater Poland Cancer Centre

**TABLE 3.** Percent of patient medical records containing a signed informed consent form

	**ICO**		**GPCC**	
Informed consent	Pts	[%]	Pts	[%]
Yes	61	89.7	94	100
No	7	10.3	0	0

ICO = Catalan Institute of Oncology; GPCC = Greater Poland Cancer Centre; Pts = patients

**TABLE 4.** Type of examination (%)

	**Digital rectal exam**	**Colonoscopy**	**Ultrasound**		**Computed Tomography**			**MRI**

			**Abdomen**	**Transrectal**	**Pelvis**	**Abdomen**	**Chest**	
ICO	100%	100	0	85.3	98.5	98.5	64.7	89.7
GPCC	100%	100	100	3.2	12.8	23.4	0	21.3

MRI = magnetic resonance imaging; ICO = Catalan Institute of Oncology; GPCC = Greater Poland Cancer Centre

## References

[1] Smith TJ, Hillner BE (2001). Ensuring quality cancer care by the use of clinical practice guidelines and critical pathways. J Clin Oncol.

[2] Shortt K, Davidsson L, Hendry J, Dondi M, Andreo P (2008). International perspectives on quality assurance and new techniques in radiation medicine: outcomes of an IAEA conference. Int J Radiat Oncol Biol Phys.

[3] Cerezo L, Ciria JP, Arbea L, Linan O, Cafiero S, Valentini V (2013). Current treatment of rectal cancer adapted to the individual patient. Rep Pract Oncol Radiother.

[4] Cionini L, Gardani G, Gabriele P, Magri S, Morosini PL, Rosi A (2007). Quality indicators in radiotherapy. Radiother Oncol.

[5] (2004). Working Group “Assicurazione di Qualità in Radioterapia” of the Istituto Superiore di Sanità, editor. [Indications for quality assurance in conformal radiotherapy in Italy].

[6] Ferlay J, Parkin DM, Steliarova-Foucher E (2010). Estimates of cancer incidence and mortality in Europe in 2008. Eur J Cancer.

[7] Malicki J, Litoborski M, Bogusz-Czerniewicz M, Swiezewski A (2009). Cost-effectiveness of the modifications in the quality assurance system in radiotherapy in the example of in-vivo dosimetry. Phys Med.

[8] Mainz J (2003). Defining and classifying clinical indicators for quality improvement. Int J Qual Health Care.

[9] Engstrom PF, Arnoletti JP, Benson AB, Chen Y-J, Choti MA, Cooper HS (2009). NCCN Clinical Practice Guidelines in Oncology: rectal cancer. J Natl Compr Canc Netw.

[10] Fortin A, Bairati I, Albert M, Moore L, Allard J, Couture C (2002). Effect of treatment delay on outcome of patients with early-stage head-and-neck carcinoma receiving radical radiotherapy. Int J Radiat Oncol Biol Phys.

[11] Chen Z, King W, Pearcey R, Kerba M, Mackillop WJ (2008). The relationship between waiting time for radiotherapy and clinical outcomes: a systematic review of the literature. Radiother Oncol.

[12] Korsgaard M, Pedersen L, Laurberg S (2008). Delay of diagnosis and treatment of colorectal cancer: a population-based Danish study. Cancer Detect Prev.

[13] Duff SE, Wood C, McCredie V, Levine E, Saunders MP, O’Dwyer ST (2004). Waiting times for treatment of rectal cancer in North West England. J R Soc Med.

[14] Fesinmeyer MD, Mehta V, Blough D, Tock L, Ramsey SD (2010). Effect of radiotherapy interruptions on survival in medicare enrollees with local and regional head-and-neck cancer. Int J Radiat Oncol Biol Phys.

[15] Bese NS, Hendry J, Jeremic B (2007). Effects of prolongation of overall treatment time due to unplanned interruptions during radiotherapy of different tumor sites and practical methods for compensation. Int J Radiat Oncol Biol Phys.

[16] Conde S, Borrego M, Teixeira T, Teixeira R, Sa A, Soares P (2013). Neoadjuvant oral vs. infusional chemoradiotherapy on locally advanced rectal cancer: prognostic factors. Rep Pract Oncol Radiother.

[17] Tremblay D, Roberge D, Cazale L, Touati N, Maunsell E, Latreille J (2011). Evaluation of the impact of interdisciplinarity in cancer care. BMC Health Serv Res.

[18] Jones WE, Thomas CR, Herman JM, Abdel-Wahab M, Azad N, Blackstock W (2012). ACR appropriateness criteria: resectable rectal cancer. Radiat Oncol.

[19] Mihaylova I, Parvanova V, Velikova C, Kurteva G, Ivanova D (2011). Degree of tumor regression after preoperative chemoradiotherapy in locally advanced rectal cancer-Preliminary results. Rep Pract Oncol Radiother.

[20] Molinari C, Ballardini M, Teodorani N, Giannini M, Zoli W (2011). Genomic alterations in rectal cancer tumors and response to neoadjuvant chemioradiotherapy; an exploratory study. Radiat Oncol.

[21] Bogusz-Osawa M, Osawa T (2005). The influence of the European and Polish acts of law, regulations and standards on the forms and the contents of the informed consent for oncological treatments. Rep Pract Oncol Radiother.

[22] Byrne DJ, Napier A, Cuschieri A (1988). How informed is signed consent?. Br Med J (Clin Res Ed).

[23] Malicki J (2012). The importance of accurate treatment planning, delivery, and dose verification. Rep Pract Oncol Radiother.

[24] Richetti A, Fogliatta A, Clivo A, Nicolin G, Pesce G (2010). Neo-adjuvant chemo-radiation of rectal cancer with volumetric modulated arc therapy: summary of technical and dosimetric features and early clinical experience. Radiat Oncol.

[25] Saminathan S, Chandraraj S, Sridhar CH, Manickam R (2012). Comparison of individual and composite field analysis using array detector for Intensity Mosulated Radiotherapy dose verification. Rep Pract Oncol Radiother.

[26] Edwards CR, Mountford PJ (2009). Characteristics of in vivo radiotherapy dosimetry. Br J Radiol.

[27] Edwards CR, Hamer E, Mountford PJ, Moloney AJ (2007). An update survey of UK in vivo radiotherapy dosimetry practice. Br J Radiol.

[28] Williams MV, McKenzie A (2008). Can we afford not to implement in vivo dosimetry?. Br J Radiol.

[29] The Royal College of Radiologists (2008). Towards safer radiotherapy.

[30] Woolf SH, Grol R, Hutchinson A, Eccles M, Grimshaw J (1999). Potential benefits, limitations, and harms of clinical guidelines. BMJ.

[31] Guedea F, Malicki J (2013). Quality indicators in radiation oncology: in regard to Albert and Das. Int J Radiat Oncol Biol Phys.

[32] Ishikura S (2008). Quality assurance of radiotherapy in cancer treatment: toward improvement of patient safety and quality of care. Jpn J Clin Oncol.

[33] Savatta SG, Temple L (2005). Quality of life in patients treated for curable rectal cancer. Semin Colon Rectal Surg.

[34] International Atomic Energy Agency (1996). International basic safety standards for protection against ionizing radiation and for the safety of radiation sources.

[35] Di Genesio Paglica M, Turri L, Munoz F, Melano A, Bacigalupo A, Franzone P (2013). Patterns of practice in the radiation therapy management of rectal cáncer: survey of the Interregional Group Piedmont, Valle d’Aosta and Liguria of the “Associazione Italiana di Radioterapia Oncologica (AIRO). Tumori.

